# Associations between short‐term exposure to fine particulate matter and acute myocardial infarction: A case‐crossover study

**DOI:** 10.1002/clc.24111

**Published:** 2023-08-04

**Authors:** Shiva Tabaghi, Mehdi Sheibani, Isa Khaheshi, Reza Miri, Mohammad Haji Aghajani, Morteza Safi, Vahid Eslami, Mehdi Pishgahi, Saeed Alipour Parsa, Mohammad Hassan Namazi, Mohammad Reza Beyranvand, Nasim Sohrabifar, Hossein Hassanian‐Moghaddam, Fatemeh Pourmotahari, Shahrzad Khaiat, Mohammad Ali Akbarzadeh

**Affiliations:** ^1^ Cardiovascular Research Center Shahid Beheshti University of Medical Sciences Tehran Iran; ^2^ Prevention of Cardiovascular Disease Research Center Shahid Beheshti University of Medical Sciences Tehran Iran; ^3^ Department of Cardiology Shahid Labbafinejad Hospital, Shahid Beheshti University of Medical Sciences Tehran Iran; ^4^ Department of Cardiology Shohada‐e Tajrish Hospital, Shahid Beheshti University of Medical Sciences Tehran Iran; ^5^ Department of Cardiology Taleghani Hospital, Shahid Beheshti University of Medical Sciences Tehran Iran; ^6^ Loghman Hakim Hospital, Shahid Beheshti University of Medical Sciences Tehran Iran; ^7^ Department of Community Medicine School of Medicine, Dezful University of Medical Sciences Dezful Iran; ^8^ University College Dublin School of Medicine Dublin Ireland

**Keywords:** acute myocardial infarction, air pollution, fine particulate matter

## Abstract

**Background:**

Previous studies evaluated the impact of particle matters (PM) on the risk of acute myocardial infarction (AMI) based on local registries.

**Hypothesis:**

This study aimed to evaluate possible short term effect of air pollutants on occurrence of AMI based on a specific case report sheet that was designed for this purpose.

**Methods:**

AMI was documented among 982 patients who referred to the emergency departments in Tehran, Iran, between July 2017 to March 2019. For each patient, case period was defined as 24 hour period preceding the time of emergency admission and referent periods were defined as the corresponding time in 1, 2, and 3 weeks before the admission. The associations of particulate matter with an aerodynamic diameter ≤2.5 μm (PM_2__.5_) and particulate matter with an aerodynamic diameter ≤10 μm (PM_10_) with AMI were analyzed using conditional logistic regression in a case‐crossover design.

**Result:**

Increase in PM_2.5_ and PM_10_ was significantly associated with the occurrence of AMI with and without adjustment for the temperature and humidity. In the adjusted model each 10 μg/m^3^ increase of PM_10_ and PM_2.5_ in case periods was significantly associated with increase myocardial infarction events (95% CI = 1.041−1.099, OR = 1.069 and 95% CI = 1.073−1.196, and OR = 1.133, respectively). Subgroup analysis showed that increase in PM_10_ did not increase AMI events in diabetic subgroup, but in all other subgroups PM_10_ and PM_2__.5_ concentration showed positive associations with increased AMI events.

**Conclusion:**

Acute exposure to ambient air pollution was associated with increased risk of AMI irrespective of temperature and humidity.

## Graphical abstract

Case crossover analysis of 982 patients with acute myocardial infarction (MI) demonstrated that Increase in PM_2__.5_ and PM_10_ were significantly associated with the occurrence of MI. PM_2__.5_ concentration showed positive associations with increased MI events in male and female, middle age and old age, diabetics and non‐diabetics, hypertensive and non‐hypertensive populations.

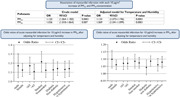

## 1 INTRODUCTION

Cardiovascular diseases particularly ischemic heart disease (IHD) is the most common cause of mortality worldwide.^1^ In addition to the common and well‐known risk factors, several epidemiological studies in the past two decades showed the effect of air pollution on cardiovascular disease and mortality.^2^, ^3^, ^4^, ^5^, ^6^, ^7^, ^8^, ^9^, ^10^ The mechanism of this association has not yet been clearly stablished. Various air pollutants are investigated including nitrogen dioxide (NO_2_), sulfur dioxide (SO_2_), Carbone monoxide (CO), and particle matters (PM).^11^, ^12^ PMs are categorized based on particle size to PM_2.5_ (PM with an aerodynamic diameter ≤2.5 μm) and PM_10_ (PM with an aerodynamic diameter ≤10 μm).^13^ Acute and chronic effects of PM_2.5_ and PM_10_ on cardiovascular system were evaluated in some studies,^14^, ^15^, ^16^ and their association with cardiovascular disease was identified.

Air pollution is a serious health concern in megacities all over the world such as Beijing, Hong Kong, Delhi, and Tehran.^17^ Tehran is the capital city of Iran with more than 8.5 million population that reaches up to 12.5 million during the daytime with people commuting for work from nearby regions. Topography of this city with the surrounding mountains prone it to air pollution.^17^, ^18^

To date the previous studies evaluating the impact of PM_2.5_ and PM_10_ on the risk of myocardial infarction (MI) have been extracted from the local registries. In this study, a specific case report sheet was designed for the same purpose, making this study the first prospective case cross‐over study to our knowledge that assesses the association between acute exposure of ambient air pollution and MI. This study was conducted in Tehran, a city with one of the highest level of air pollution in Iran.

## 2 METHODS

### 2.1 Study population

**Figure 1:**
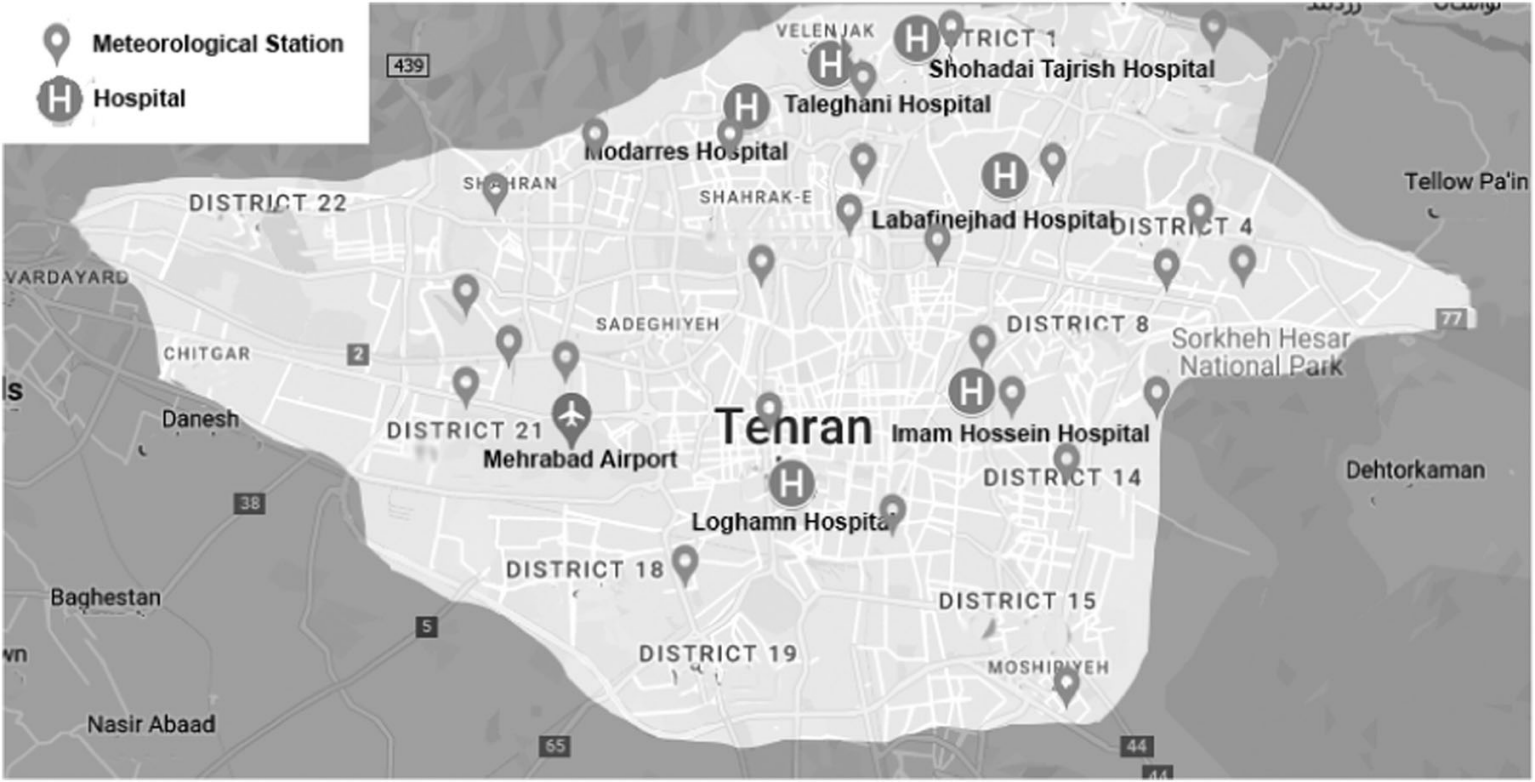
Map of Tehran and localization of included hospitals and meteorological stations.

This study was conducted in six general hospitals affiliated with Shahid Beheshti University of Medical Sciences (SBMU), in different locations of Tehran (Figure 1). The study protocol was approved by institutional review board and the ethics committee of SBMU.

From July 2017 to March 2019, all of the patients with angina or angina equivalent symptoms referred to the emergency department with an elevated troponin I level (value above the 99th percentile of the upper reference level) and diagnosed as acute myocardial infarction (AMI) were included.^19^ Patients with any other causes of elevated troponin I such as sepsis, decompensated heart failure, severe renal failure, rhabdomyolysis, and pulmonary embolism were excluded.^20^ Also, patients who had traveled outside of the Tehran city any time in the last 1 month before their presentation or who had been admitted due to acute coronary syndrome during this period were excluded. The case report sheet designed specifically for this study and approved by the scientific board includes the demographic data, patient residence information, risk factors, and inpatient lab data filled by trained nurses under the supervision of a cardiologist at each hospital.

### 2.2 Air pollution measurement

In Tehran, ambient pollutant levels are continuously monitored at more than 20 air quality monitoring stations across the city meteorological stations. Daily average concentration of PMs measured with the method of beta radiation absorption and using Geiger−Müller counter in two sizes of less than 10 μm and less than 2.5 μm were obtained from the nearest meteorological station to the patients' living place, reported by the Tehran Air Quality Company (http://airnow.tehran.ir/home/DataArchive.aspx) (Figure 1). To control potential confounding meteorologic parameters, the mean daily humidity (%) and temperature (°C) were obtained from Tehran station of Meteorological Organization (Mehrabad station).

Ambient air pollutant exposure and meteorological data were analyzed by mean exposure in the last 24 hours, 1, 2, and 3 weeks before the day of emergency department presentation.

### 2.3 Statistical analysis

In a case‐crossover design, for the patients referred to the emergency department with the impression of acute MI air pollutant concentrations at the index period (case period) were compared with air pollutant concentrations at comparable controls, or referent times. Case period was defined as 24 hour period preceding the time of emergency admission. Referent period was defined as the corresponding time in 1, 2, and 3 weeks before the admission. In other words, for each patient every case period has 3 referent periods to compare with. The mean level for each air pollution parameter was calculated at the case and referent periods, separately. Choosing the referent periods closer to the index period reduces the various time‐dependent changes in individual characteristics or seasonal risk factors. Also, selecting the same day of the week controls for the possibility of day‐of‐week effects.

Correlation between levels of air pollutants and meteorological parameters was measured (or evaluated) with Spearman's correlation coefficient. Conditional logistic modeling was applied data with 1 case (case period) + 3 controls (3 referent periods) per set to calculate odds ratios and 95% confidence intervals.

First, crude conditional logistic regression models were used to evaluate the association between each air pollutant exposure and MI. Subsequently, meteorological factors such as temperature and humidity were adjusted as the covariates in all of the models. PM_2.5_ and PM_10_ were entered into the models as continuous variables. The odds ratio values were calculated for each 10 μg/m^3^ increase in air pollutant exposure.

To evaluate whether clinical and demographic factors could contribute to variability in odds ratio of MI, a subcohort case‐crossover analysis was conducted stratified by sex, age ≤65 versus >65, diabetes mellitus, and hypertension as modifiers.

All data management and analyses were performed using R (version: 4.0.5). Continuous variables were presented as mean ± standard deviation. *p*‐value < .05 was set as statistically significant.

### 2.4 Ethical consideration

This study was conducted in accordance with the Declaration of Helsinki and the study protocol approved by the Institutional Review Board (IRB) at the National Center for Mental Health, Seoul, Korea (IRB number: 116271‐1027‐57).

## 3 RESULTS

Totally, 982 qualified individuals who experienced acute MI were enrolled, of these, 228 (23.2%) were female. The mean age of the patients was 60.79 ± 12.76 (range 20−98 years). The demographic and clinical features of patients are shown in Table 1.

**Table 1:** Basic demographic and clinical characteristics of the patients with myocardial infarction.

Parameter	Number (%)
Sex (female)	228 (23.2)
Age >65 years	336 (34.39)
Smoker	376 (38.3)
Diabetic	273 (27.8)
Hypertensive	394 (40.1)

The daily average concentrations for PM_2.5_ and PM_10_ 24 hours before MI were 32.59 ± 16.83 and 82.96 ± 33.47 µg/m,^3^ respectively. Also, the average daily temperature, and relative humidity of the day before MI were 20.22 ± 9.32 (°C) and 31.67 ± 16.16%, respectively. Summary characteristics of fine particles, temperature, and humidity during the study period are shown in Table 2.

**Table 2:** Characteristics of air pollution and meteorological parameters during the study period (*n* = 638 days).

Parameter	Mean ± SD
Temperature (°C)	18.47 ± 10.04
Humidity (%)	33.82 ± 16.64
PM_2.5_	21.90 ± 9.14
PM_10_	56.66 ± 24.33

Abbreviation: SD, standard deviation.

The PM_10_ had a very weak positive correlation with temperature and a weak inverse correlation with humidity, and the PM_2.5_ had a very weak inverse correlation with temperature and a very weak positive correlation with humidity. There was also a strong positive correlation between PM_10_ and PM_2.5_ (*r* = .716), and a very strong inverse correlation between temperature and humidity (*r* = −.832) (Supporting Information: Table 1).

Analysis of the data revealed that increase in PM_2.5_ and PM_10_ were significantly associated with the occurrence of MI with and without adjustment for the temperature and humidity. In the adjusted model, it has been shown that 10 μg/m^3^ increase of PM_10_ in case period was significantly associated with increased MI events (*p* = .0001, with 95% CI = 1.041−1.099, and OR = 1.069). Also, 10 μg/m^3^ increase in PM_2.5_ was significantly associated with increased MI events (*p* = .0001, 95% CI = 1.073−1.196, and OR = 1.133) (Table 3).

**Table 3:** Association of myocardial infarction with each 10 μg/m^3^ increase of PM_2.5_ and PM_10_ concentration.

Pollutants	Crude model			Adjusted model^a^		
	OR	95% CI	*p* Value	OR	95% CI	*p* Value
PM_2.5_	1.122	(1.064−1.183)	.0001	1.133	(1.073−1.196)	.0001
PM_10_	1.036	(1.010−1.064)	.007	1.069	(1.041−1.099)	.0001

Abbreviations: CI, confidence interval; OR, odds ratio.

^a^ Adjusted for temperature and humidity.

Subgroup analysis was performed after stratification by individual characteristics. It showed that an increase in PM_10_ did not increase MI events in diabetic subgroup, but in all other subgroups PM_10_ and PM_2.5_ concentration showed positive associations with increased MI events (Table 4 and Supporting Information: Figure 1, 2).

**Table 4:** Association of myocardial infarction occurrence for each 10 μg/m^3^ increase of exposure to PM_2.5_, PM_10_ after adjusting for temperature and humidity in different subgroups.

	PM_2.5_		PM_10_	
	OR (95% CI)	*p* Value	OR (95% CI)	*p* Value
Female	1.166 (1.042−1.306)	.007	1.097 (1.038−1.161)	.001
Male	1.123 (1.056−1.195)	.0002	1.061 (1.028−1.094)	.0001
Age ≤65	1.169 (1.078−1.268)	.0001	1.070 (1.028−1.114)	.0008
Age >65	1.105 (1.026−1.190)	.007	1.070 (1.031−1.111)	.0002
Smoker	1.212 (1.105−1.330)	.000	1.084 (1.037−1.134)	.0003
Nonsmoker	1.121 (1.045−1.202)	.001	1.073 (1.036−1.112)	.000
Diabetic	1.124 (1.020−1.239)	.018	1.041 (0.992−1.094)	.101
Non‐diabetic	1.139 (1.066−1.218)	.0001	1.085 (1.050−1.122)	.000
Hypertensive	1.129 (1.043−1.223)	.002	1.060 (1.017−1.105)	.004
Non‐hypertensive	1.138 (1.055−1.228)	.0007	1.078 (1.039−1.119)	.000

## 4 DISCUSSION

This case cross‐over study evaluated the effect of short‐term exposure to ambient PM_2.5_ and PM_10_ on the risk of MI. Given the proven effect of humidity and temperature on air pollution, we also adjusted these parameters to eliminate their confounding effect on the final results. This investigation showed that short‐term exposure to ambient PM_2.5_ and PM_10_ was associated with increased risk of MI in both crude and adjusted models.

Several underlying mechanisms, such as the systemic inflammation caused by oxidative stress, vascular dysfunction and remodeling, increased arterial stiffness, blood pressure, pro‐thrombotic pathway activation, and increased fibrinolysis have been suggested to explain the effect of acute exposure to PMs and other air pollutants on MI events.^21^, ^22^, ^23^ PMs can disrupt endothelial cellular hemostasis and trigger formation of free reactive oxygen species (ROS) by mitochondria. PMs are also deposited in the lung and phagocytosed by the alveolar macrophages. Overactivation of these macrophages and also cellular/tissue damage caused by ROS overproduction lead to local and systemic inflammation.^23^ These proinflammatory states and oxidative stress may play a major role in atherosclerotic plaque vulnerability and hyper coagulopathy state.^24^ Animal studies revealed that acute exposure to air pollutants can promote atherosclerotic plaque growth with increased vulnerability for plaque rupture.^25^, ^26^ Plaque rupture may then lead to MI.

Several studies were performed to prove the effect of air pollutants on the risk of MI. In a time series study, Yu et al. detected a 1.63% and 0.80% increase in daily MI incidence reported with 10 μg/m^3^ increments in concentration of PM_2.5_ and PM_10_, respectively.^27^ However, in a multicity time‐series analysis, Stieb et al. did not find significant correlation between PM particles exposure and elevated risk of MI.^28^ To control for the confounding factors, most recent studies adapted a case‐crossover design.

In a recent case‐crossover study, Kim et al. reported that the short‐term exposure to PM_10_ has no association with IHD with a positive association seen in long term exposures.^22^ They enrolled patients with IHD with no acute MI and their short‐term exposure was defined as 30 days before the index day of admission. These differences may explain for their different results. In another case‐crossover study, Salivan et al. did not report any significant association between short‐term PM_10_ exposure and acute MI.^29^ Some other studies also demonstrated no significant relationship between PM_10_ and acute MI.^24^, ^30^, ^31^, ^32^, ^33^

On the other hand and consistent with our results, Huang et al. reported a positive association between PM_10_ exposure and slightly higher risk of ACS. For each 10 μg/m^3^ increment in PM_10_, a 3.7% increase in the risk of ACS was reported.^34^ Some other studies also noted the association between PM_10_ exposure and acute MI.^35^, ^36^, ^37^, ^38^

In many studies, the role of PM_2.5_ exposure on occurrence of MI was more prominent than PM_10_.^39^, ^40^, ^41^, ^42^ In a meta‐analysis of 31 studies by Luo et al., the association between PM_2.5_ and MI had higher odds ratio than PM_10_.^43^ Similarly, our findings showed that PM_2.5_ exposure was associated with MI at a higher odds ratio compared to PM_10_ exposure. As PM_2.5_ comprises a fraction of PM_10_, the role of PM_10_ in acute MI events may be due to the greater effect of PM_2.5_ not masking the true effect of particles between 2.5 and 10 μm. Therefore, these studies cannot prove the independent role of PM_10_ in the incidence of MI.

As mentioned, the association between PM_2.5_ exposure and MI has been shown in this study and many other studies.^27^, ^35^, ^36^, ^37^, ^38^, ^40^, ^41^, ^42^, ^43^ A recent meta‐analysis demonstrated that a 10 μg/m^3^ increase in PM_2.5_ was associated with about 2% increase in risk of MI.^44^ In our study, for every 10 μg/m^3^ increase in PM_2.5_ and PM_10_ concentration, the incidence of MI increased by 13% and 6.9%, respectively.

Argacha et al. showed that 10 μg/m^3^ increase in PM_2.5_ within the 24 h before the event was associated with 2.8% increased risk of ST elevation MI. However, they didn't find similar correlation between PM_2.5_ concentration and non‐ST elevation MI.^45^ Triggering of transmural infarction, but not non‐transmural infarction, by ambient fine particles was also reported by Rich et al.^46^

This study was conducted in Tehran, one of the most polluted cities in the world. Yen et al. explained short‐term air pollutant exposure has a positive correlation with the severity of pollution in the study region. Therefore, in the areas with moderate or high air pollution, short‐term pollutant exposure increases the incidence of acute MI, but in the areas with lower pollution, short term exposure does not change the risk of MI.^47^ This may explain the difference found between our results and some other studies,^32^, ^48^ and the higher percentage of increased MI risk in our study compared to other similar studies.^27^, ^35^, ^37^, ^40^

In some studies the impact of air pollution on the incidence of IHD varies in different demographic subgroups.^22^, ^49^ Our data showed an overall increased risk of MI for each 10 μg/m^3^ increase in both PM_2.5_ and PM_10_ in both men and women, smoker and nonsmoker, hypertensive and non‐hypertensive, and age subgroups. Several previous studies similarly revealed increased risk of MI incidence due to air pollution in different demographic subgroups.^22^, ^38^, ^45^, ^49^, ^50^ Interestingly, we found that diabetic patients don't have any significant increased risk of MI with increasing PM_10_ concentration. Same results were reported in another study in relation to patients with diabetes.^6^ Unlike our study, some other investigations showed that exposure to higher concentrations of PM_10_ was directly correlated with increased MI events in type 2 diabetes patients.^6^, ^45^, ^51^ Diabetes Mellitus is a major risk factor for MI and a significant percentage of patients with DM are presented with coronary artery atherosclerosis. This may explain why in our study increase in PM_10_ concentration did not have enough power to increase the risk in this subgroup significantly.

The key strength of this study is that it is the only prospective case‐crossover study to our knowledge to measure the effect of ambient air pollutions on acute MI. However, some limitations must be considered. First, Tehran is one of the most crowded and polluted cities in the world and its meteorological finding cannot be generalized to other places. Second, the level of patient exposure to pollutants cannot be directly measured. Exposures were determined and recorded based on the time spent outdoors and/or use of personal protective equipment in each patient. Finally, this study has missing data from the patients presented with lethal MI and out of hospital cardiac arrest.

## 5 CONCLUSION

Short term exposure to PMs was associated with increased risk of MI. This effect was independent of humidity and temperature in adjusted model and presented in all study subgroups except patients with diabetes mellitus. PM_10_ was not significantly increased MI events in diabetics.

## CONFLICT OF INTEREST STATEMENT

The authors declare no conflict of interest.

## Supporting information

Supporting information.

## Data Availability

The data that support the findings of this study are available on request from the corresponding author upon reasonable request and with the permission of Tehran Air Quality Company and Iran Meteorological Organization. The data are not publicly available due to privacy or ethical restrictions. All authors take responsibility for all aspects of the reliability and freedom from bias of the data presented and their discussed interpretation.
