# Supplementary material for: Associations between short‐term exposure to fine particulate matter and acute myocardial infarction: A case‐crossover study
Source: Clin Cardiol. 2023 Aug 4;46(11):1319–25. doi: 10.1002/clc.24111 (PMC10642339; doi:10.1002/clc.24111)
Supplement: Supplementary file 1 — Supporting information. [file CLC-46-1319-s001.docx]

**Supplementary Table 1**: Spearman’s correlation coefficients between air pollutants and meteorological parameters

| Humidity | Temperature | PM_10_ | PM_2.5_ |  |
| --- | --- | --- | --- | --- |
|  |  |  | 1 | **PM_2.5_** |
|  |  | 1 | 0.716 | **PM_10_** |
|  | 1 | 0.166 | -0.200 | **Temperature** |
| 1 | -0.832 | -0.258 | 0.068 | **Humidity** |

*All pairwise correlation coefficients were statistically significant (P-value <0.05).

**Supplementary Fig. 1**. Odds ratios of acute myocardial infarction for 10 μg/m3 increase in PM_2.5_ after adjusting for temperature and humidity stratified by various characteristics.

**Supplementary Fig 2.** Odds ratios of acute myocardial infarction for 10 μg/m3 increase in PM_10_ after adjusting for temperature and humidity stratified by various characteristics.
